# Blood pressure and resting heart rate in 3-17-year-olds in Germany in 2003–2006 and 2014–2017

**DOI:** 10.1038/s41371-021-00535-2

**Published:** 2021-04-14

**Authors:** Giselle Sarganas, Anja Schienkiewitz, Jonas D. Finger, Hannelore K. Neuhauser

**Affiliations:** 1 Department of Epidemiology and Health Monitoring, Robert Koch Institute, Berlin, Germany; 2 DZHK (German Centre for Cardiovascular Research), partner site Berlin, Berlin, Germany

**Keywords:** Cardiovascular diseases, Risk factors

## Abstract

To track blood pressure (BP) and resting heart rate (RHR) in children and adolescents is important due to its associations with cardiovascular outcomes in the adulthood. Therefore, the aim of this study was to examine BP and RHR over a decade among children and adolescents living in Germany using national examination data. Cross-sectional data from 3- to 17-year-old national survey participants (KiGGS 2003–06, *n* = 14,701; KiGGS 2014–17, *n* = 3509) including standardized oscillometric BP and RHR were used for age- and sex-standardized analysis. Measurement protocols were identical with the exception of the cuff selection rule, which was accounted for in the analyses. Different BP and RHR trends were observed according to age-groups. In 3- to 6-year-olds adjusted mean SBP and DBP were significantly higher in 2014–2017 compared to 2003–2006 (+2.4 and +1.9 mm Hg, respectively), while RHR was statistically significantly lower by −3.8 bpm. No significant changes in BP or in RHR were observed in 7- to 10-year-olds over time. In 11- to 13-year-olds as well as in 14- to 17-year-olds lower BP has been observed (SBP −2.4 and −3.2 mm Hg, respectively, and DBP −1.8 and −1.7 mm Hg), while RHR was significantly higher (+2.7 and +3.7 bpm). BP trends did not parallel RHR trends. The downward BP trend in adolescents seemed to follow decreasing adult BP trends in middle and high-income countries. The increase in BP in younger children needs confirmation from other studies as well as further investigation. In school-aged children and adolescents, the increased RHR trend may indicate decreased physical fitness.

## Introduction

To track blood pressure (BP) and resting heart rate (RHR) in children and adolescents is important due to its associations with cardiovascular outcomes in the adulthood. BP in childhood correlates with BP in adulthood [1], hence children with elevated BP have a higher probability of developing hypertension in adulthood than children with lower BP. High compared to low RHR was related to a higher risk for all-cause mortality in a recent meta-analysis [2]. Therefore, population-based monitoring of SBP and RHR time trends is necessary. Yet, far less studies exist on SBP and RHR time trends in children compared to adults because only few countries have repeated national health examination surveys with BP and RHR measurements in children and adolescents in place [3, 4]. When analyzing BP trends in an adult population, mean SBP is considered an important indicator of BP-associated risk because it captures not only BP elevations above the fixed hypertension threshold but the whole BP distribution including moderately elevated BP. Even a BP below the hypertension threshold is still associated with increased cardiovascular risk [5]. Despite the strong association between body-mass-index (BMI) and BP [6–9], available worldwide data on mean SBP in children, which has been summarized in several reviews [10–12], showed a rather consistent decrease in mean SBP despite the obesity epidemic. However, there are some recent exceptions, i.e., fluctuating, stagnating, or increasing mean SBP in China [4, 9, 13–15] and increasing BP in selected pediatric age groups in UK [16]. The aim of this study was to examine BP and RHR over a decade among children and adolescents living in Germany using national examination data.

## Methods

### Study design and study population

The German Health Interview and Examination Survey for Children and Adolescents (KiGGS) is a nationwide study based on a stratified population registry sample. It is a part of the Federal Health Monitoring System operated by the Robert Koch Institute and includes repeated cross-sectional surveys (examination and interview) of children and adolescents between 0 and 17 years of age that are representative for the German population [17, 18]. In the KiGGS Baseline study (2003–06), children and adolescents aged 0–17 years were interviewed and examined (response rate 67%) and had BP and RHR measurements from age 3 years (*n* = 14,835) [18]. KiGGS Wave 2 was the second national health examination survey in children and adolescents aged 0–17 years in Germany in 2014–17 and had a response rate of 40.1% [17]. BP and RHR measurements were available for 3567 participants aged 3–17 years.

BP measurement methods were standardized and followed the same protocol, with the exception of cuff selection rule, in KiGGS Baseline study 2003–06 and KiGGS Wave 2 2014–17. At both time-points, BP measurements were taken in the sitting position on a height adjustable chair with a backrest, the right forearm resting on a table at the level of the heart, the elbow slightly bent, the legs uncrossed, and the feet placed firmly on the floor. Four cuff bladders (6 × 12; 9 × 18; 12 × 23, or 17 × 38.6 cm^2^) were available for the following arm circumferences 10.0–17.9 cm, 18.0–24.9 cm, 25.0–32.9 cm and 33.0–47.0 cm, respectively. In KiGGS Baseline an older rule for cuff selection was followed to cover at least two-thirds of the upper arm length as measured from the axilla to the antecubital fossa, while in KiGGS Wave 2 arm circumferences measures were followed for cuffs selection. As the cuff size selection based on arm length has been shown to lead to wider cuffs and therefore to lower BP than the selection based on covering at least 40% of the arm circumference [19], we have adjusted for the cuffs in the analysis. Two readings of SBP, diastolic blood pressure (DBP), mean arterial BP, and RHR were obtained, after a non-strenuous part of the examination and an additional five-minute rest in both surveys, at a two-minute interval with an automated upper armoscillometric device (DatascopeAccutorr Plus, Mahwah, NJ), which was previously validated in children aged 5–15 years according to the international protocol of the European Society of Hypertension (ESH) [20, 21]. The mean of the two SBP and DBP measurements was used for this analysis. BP in children is age, gender, and height dependent; therefore, national reference percentiles based on KiGGS data from non-overweight children were used [20]. The proportion of BP measures classified according to the international definitions as normal, elevated, and hypertensive were calculated for the KiGGS Wave 2 population according to the ESH Hypertension Definition 2016: [22] for children and adolescents <15 years old normal BP: SBP and DBP < 90th percentile; elevated BP: SBP or DBP ≥ 90th to <95th percentile and hypertensive BP: SBP or DBP ≥ 95th percentile. For adolescents 16 years and older normal BP: < 130/85 mm Hg; elevated BP: 130–139 / 85–89 mm Hg and hypertensive BP ≥ 140/90 mm Hg and according to the American Academy of Pediatrics (AAP) 2017 definition [23] for children and adolescents <13 years old normal BP: SBP and DBP < 90th percentile; elevated BP: SBP or DBP ≥ 90th to <95th percentile and hypertensive BP: SBP or DBP ≥ 95th percentile. For adolescents 13 years and older normal BP: < 120/80 mm Hg; elevated BP: 120–129 / <80 mm Hg and hypertensive BP ≥ 130/80 mm Hg.

Height was measured at both time points without shoes to the nearest 0.1 cm by using portable stadiometer devices (in KIGGS Baseline: Holtain Ltd., UK and in KIGGS Wave 2: seca 274, Fa. seca, Hamburg, Germany) and weight in underwear to the nearest 0.01 kg with a calibrated scale (seca mBCA 515/514; Fa. seca, Hamburg, Germany). A person’s BMI was calculated from the ratio between body weight and height squared (kg/m^2^). Up to the age of 18, BMI percentile curves were applied taking into account age and gender. In Germany, overweight and obesity are usually defined by applying national reference percentiles according to Kromeyer-Hauschild et al. [24]. Children with a BMI above the 90th percentile are considered overweight and obesity is defined as a BMI above the 97th percentile. Sensitivity analyses with the International Obesity Task Force (IOTF) [25] classification were also performed.

“Organized sport participation” was assessed among children aged 3–10 years based on self-reports provided by their parent in proxy interviews. At KiGGS Baseline and KiGGS Wave 2, the parents were asked whether their child participates in sports or exercise in sport clubs. A dichotomous variable was constructed: organized sports participation, yes or no.

“Self-rated physical fitness” level was assessed among children and adolescents aged 11–17 years at KiGGS Baseline and KiGGS Wave 2 with the following question: “How would you rate your own physical fitness level?” “Very good”; “Good”; “Intermediate”; “Bad”; “Very bad”. A dichotomous variable was constructed: Very good/good self-rated physical fitness, yes or no.

Birth weight (in g) was reported by the parents and we have classified it into <2500 g, as low birth weight, normal birth weight between 2500 and <4000 g and high birth weight > = 4000 g.

### Statistical analysis

Cross-sectional data from 3- to 17-year-olds from KiGGS Baseline 2003–06 (*n* = 14,701) and from KiGGS Wave 2 2014–17 (*n* = 3509) were analyzed. In the descriptive analysis, proportions and mean values with 95% confidence intervals (95% CI) differentiated according to age group and survey period were calculated. We have investigated mean BP and RHR over time and factors influencing changes among the different age groups. The combined dataset of the two surveys was used for linear regression models with *z*-scores of SBP, DBP, and RHR, respectively, as dependent variables. We included a “survey” variable as independent variable to denote the survey time period to test time trends for statistical significance. Differences in trends across subgroups were tested by adding interaction terms to the models (sex/age × survey). Accounting for the different rule applied for cuff selection in the two surveys (Table 1), we have included cuff size as a covariate for estimating the adjusted BP trend in time. Other included covariates in the multivariate models were sex, BMI, height, physical activity metrics, RHR, and low birth weight. A *p* < 0.05 was defined as statistically significant. All analyses were performed with STATA SE 15 using adaptive weighting factors and survey procedures to adjust for the cluster design. Adjustment weights considered deviation between the study sample and the population structure at the time of survey (31st December, 2015) according to age, sex, region, and parental education and nationality. Using sampling weights with the same age- and sex distribution for both waves is equivalent to standardizing to the same population and allows comparisons over time independent of demographic changes.

**Table 1:** Baseline characteristics of cross-sectional study participants (2003–2006 and 2014–2017) weighted to the population in Germany as of 31.12.2015.

	3–6 years			7–10 years			11–13 years			14–17 years		
Characteristics	2003–2006 (*n* = 3764)	2014–2017 (*n* = 840)	*p*-trend	2003–2006 (*n* = 4134)	2014–2017 (*n* = 900)	*p*-trend	2003–2006 (*n* = 3073)	2014–2017 (*n* = 814)	*p*-trend	2003–2006 (*n* = 3730)	2014–2017 (*n* = 955)	*p*-trend
Mean age^a^	4.5 (4.5–4.6)	4.6 (4.5–4.6)	0.774	8.5 (8.5–8.5)	8.5 (8.4–8.6)	0.992	12.0 (11.9–12.0)	12.0 (11.9–12.1)	0.978	15.5 (15.5–15.6)	15.5 (15.4–15.6)	0.995
Female^b^	48.1 (46.3–50.0)	49.3 (45.0–53.7)	0.630	48.6 (46.7–50.4)	49.1 (45.0–53.3)	0.809	48.6 (46.5–50.7)	48.5 (44.0–53.1)	0.976	48.1 (46.2–49.9)	48.1 (43.9–52.3)	0.989
Height percentile^a^	49.5 (48.4–50.6)	47.4 (44.9–50.0)	0.145	48.5 (47.5–49.6)	49.4 (46.9–51.8)	0.550	50.1 (48.8–51.3)	49.9 (47.3–52.6)	0.922	49.5 (48.5–50.6)	50.8 (48.4–53.1)	0.345
SBP (mmHg)^a^	98.6 (98.3–98.9)	102.5 (101.8–103.2)	<0.001	103.4 (103.1–103.8)	105.6 (104.9–106.3)	<0.001	110.6 (110.1–111.0)	110.9 (110.2–111.6)	0.415	117.9 (117.5–118.3)	116.2 (115.4–117.0)	<0.001
DBP (mmHg)^a^	60.2 (60.0–60.5)	63.7 (63.0–64.3)	<0.001	63.2 (63.0–63.5)	64.7 (64.1–65.4)	<0.001	66.2 (65.9–66.5)	66.6 (66.0–67.2)	0.266	69.9 (69.6–70.1)	69.0 (68.5–69.7)	0.007
Resting heart rate^a^	94.5 (94.0–94.9)	93.9 (92.8–94.9)	0.304	82.3 (82.0–82.8)	83.4 (82.5–84.4)	0.046	78.6 (78.1–79.1)	80.9 (79.8–81.9)	<0.001	74.3 (73.8–74.7)	76.3 (75.4–77.3)	<0.001
Cuff bladder 6 × 12 cm^2^	14.7 (13.4–16.1)	67.4 (63.1–71.4)	< 0.001	0.2 (0.1–0.3)	15.2 (12.5–18.4)	< 0.001	0.1 (0.0–0.6)	1.0 (0.4–2.4)	< 0.001	0.1 (0.0–0.2)	0.1 (0.0–0.8)	< 0.001
9 × 18 cm^2^	71.5 (69.7–73.2)	32.6 (28.6–36.9)		18.3 (16.9–19.7)	75.0 (71.1–78.5)		2.2 (1.6–3.0)	62.7 (58.1–67.2)		0.2 (0.1–0.3)	26.6 (23.1–30.4)	
12 × 23 cm^2^	13.6 (12.4–15.0)	0		76.0 (74.4–77.5)	9.8 (7.4–12.8)		49.1 (47.0–51.39)	34.8 (30.4–39.5)		20.1 (18.7–21.6)	65.9 (61.8–69.8)	
17 × 39 cm^2^	0.2 (0.1–0.6)	0		5.6 (4.8–6.5)	0		48.5 (46.4–50.7)	1.4 (0.7–2.8)		79.7 (78.2–81.1)	7.4 (5.4–10.1)	
BMI (kg/m^2^)^a^	15.8 (15.8–15.9)	15.7 (15.6–15.8)	0.119	17.4 (17.3–17.5)	17.3 (16.9–17.5)	0.263	19.9 (19.8–20.1)	19.8 (19.5–20.2)	0.556	22.1 (21.9–22.2)	22.1 (21.7–22.5)	0.842
Underweight^b^	5.4 (4.6–6.3)	5.3 (3.9-7.2)	0.419	7.9 (6.9–8.9)	8.7 (6.6–11.4)	0.641	8.0 (7.0–9.2)	8.1 (6.2–10.6)	0.707	7.0 (6.1–8.0)	8.6 (6.4–11.4)	0.655
Normal-weight^b^	84.8 (83.4–86.1)	85.9 (82.5–88.6)		76.0 (74.4–77.6)	75.6 (71.7–79.2)		72.7 (70.7–74.6)	71.3 (66.8–75.5)		75.7 (74.1–77.3)	74.0 (70.0–77.6)	
Overweight^b^	6.5 (5.6–7.5)	6.9 (4.9–9.8)		9.0 (8.0–10.1)	9.8 (7.4–12.9)		11.3 (10.1–12.8)	13.3 (10.1–17.3)		8.8 (7.8–9.9)	8.9 (6.7–11.8)	
Obese^b^	3.3 (2.6–4.1)	1.9 (1.0–3.6)		7.1 (6.2–8.2)	5.9 (4.1–8.3)		8.0 (6.8–9.3)	7.3 (5.0–10.5)		8.5 (7.5–9.7)	8.5 (6.4–11.3)	
Organized sports participation (yes)^b^	51.3 (49.3–53.2)	49.3 (44.8–53.8)	0.431	68.6 (66.8–70.3)	67.8 (63.6–71.7)	0.742	–	–	–	–	–	–
(Very) good self-rated physical fitness^b^	–	–	–	–	–	–	70.9 (68.9–72.8)	71.9 (67.5–75.9)	0.688	61.9 (60.1–63.7)	63.9 (59.8–67.9)	0.376
Low birthweight (<2500 g)^b^	6.3 (5.4–7.3)	7.8 (5.6–10.9)	0.266	5.7 (4.9–6.7)	8.3 (6.2–11.1)	0.073	5.9 (4.9–7.0)	7.2 (5.1–10.2)	0.545	5.5 (4.7–6.5)	5.9 (4.1–8.2)	0.721
Current anti-hypertensive medication use	*n* = 2	*n* = 3	**–**	*n* = 4	*n* = 0	–	*n* = 2	*n* = 3	–	*n* = 9	*n* = 2	–

*p*-trend: statistical difference between time points.

^a^Mean (95% CI).

^b^Proportion (95% CI).

## Results

The characteristics of study participants in KiGGS Baseline (2003–06; *n* = 14,701) and in KiGGS Wave 2 (2014–17; *n* = 3509) are presented in Table 1. The distribution of height, BMI, low birth weight, and physical activity metrics available in both waves (organized sports participation for younger children and self-rated physical fitness for older children) did not change between the two time points. Although identical cuffs of four different sizes were available in both surveys, the cuff selection rules differed, leading to narrower cuffs on average in KiGGS Wave 2 compared to baseline (Table 1).

**Fig. 1:**
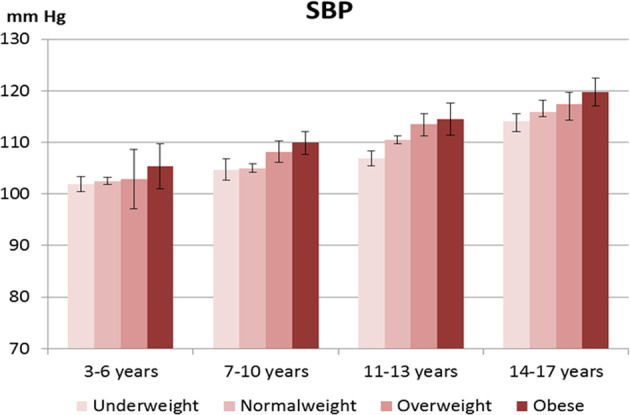
Systolic blood pressure (SBP) in KIGGS Wave 2. Mean systolic blood pressure (SBP) in mm Hg in children and adolescents from the KIGGS Wave 2 (2014–17) according to age group and body mass index (BMI) category.

**Fig. 2:**
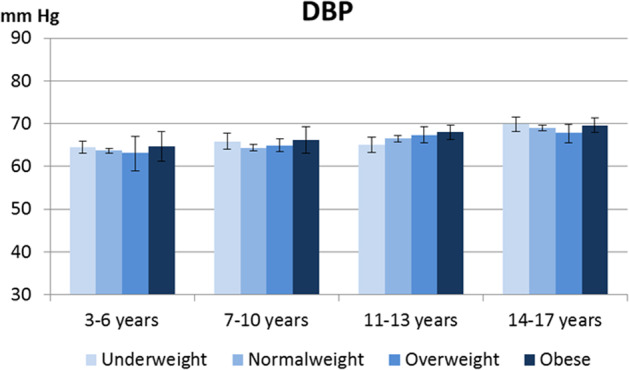
Diastolic blood pressure (DBP) in KIGGS Wave 2. Mean diastolic blood pressure (DBP) in mm Hg in children and adolescents from the KIGGS Wave 2 (2014–17) according to age group and body mass index (BMI) category.

Figures 1 and 2 illustrates the mean SBP and DBP in children and adolescents from the KiGGS Wave 2 stratified by age-group and by BMI categories. There were no significant sex differences in all age-groups and BMI categories except for SBP in normal weight age group 14–17 years (SBP males 118.8 mm Hg; 95% CI 117.5–120.1 vs. females 112.9 mm Hg; 95% CI 111.9–114.0; *p* < 0.001). Supplementary Fig. 1 and 2 illustrate the proportion of children and adolescents from the KiGGS Baseline and Wave 2 with normal, elevated, and hypertensive measures according to ESH 2016 and AAP 2017 definitions. The total proportion of children and adolescents with a hypertensive BP measure, measured on a single occasion, was 8.4% and 13.1% according to ESH 2016 and AAP 2017 definitions, respectively, in KiGGS Baseline and 10.4% and 15.6% in KiGGS Wave 2.

### BP changes

BP estimates differed between age-groups, as statistically significant interaction term between survey and age-group were observed in linear regression models. Therefore, we fitted separate models for the different age-groups. In 3- to 6-year-olds mean SBP has statistically significantly increased by +2.5 mm Hg (adjusted + 2.4 mm Hg) (*p* < 0.001) and mean DBP by +2.0 mm Hg (adjusted + 1.9 mm Hg) (*p* < 0.001). This change was confirmed in the *z*-score models by adjusting for the covariates (Tables 2 and 3). In 7- to 10-year-olds there were no significant changes in the adjusted SBP or in DBP (in mm Hg) over time; however, in the SBP z-score model, due to the covariate effect, a small but statistically significant decrease in SBP was observed. In 11- to 13-year-olds adjusted mean SBP and DBP have statistically significantly decreased by −2.4 and −1.8 mm Hg, respectively. This trend was confirmed in the *z*-score models. Likewise, in 14- to 17-year-olds adjusted mean SBP and DBP have statistically significantly decreased by −3.2 and by −1.7 mm Hg, respectively; which was confirmed with the *z*-score models.

**Table 2:** Factors associated with mean SBP *z*-score from linear regression models in 3–6-, 7–10-, 11–13-, and in 14–17-year-olds.

Age group	3–6 years			7–10 years		
	Coeff.	95% CI	*p* value	Coeff.	95% CI	*p* value
Wave 2 (Survey) adjusted^a^	0.255	0.129 to 0.382	<0.001	0.020	−0.089 to 0.129	0.716
Wave 2 (Survey) adjusted^b^	0.263	0.139 to 0.387	<0.001	−0.146	−0.257 to −0.035	0.010
Female	−0.028	−0.094 to 0.038	0.397	−0.025	−0.084 to 0.033	0.395
BMI percentile	0.009	0.008 to 0.010	<0.001	0.011	0.010 to 0.013	<0.001
Height percentile	0.001	0.000 to 0.003	0.046	0.001	−0.000 to 0.002	0.087
Sport participation (yes)	−0.076	−0.140 to −0.011	0.021	−0.042	−0.111 to 0.028	0.236
RHR (*z*-Score)	0.263	0.231 to 0.294	<0.001	0.262	0.229 to 0.294	<0.001
Birth weight						
Low (<2,5 kg)	0.408	0.265 to 0.552	<0.001	0.089	−0.030 to 0.208	0.032
High (≥4,0 kg)	−0.129	−0.236 to −0.021		−0.122	−0.233 to −0.010	
Cuff size						
Medium	−0.521	−0.631 to −0.411	<0.001	−0.970	−1.305 to −0.634	<0.001
Large	−0.667	−0.812 to −0.522		−1.374	−1.719 to −1.030	
X-Large	0.076	−0.758 to 0.910		−1.510	−1.878 to −1.142	

Age group	11–13 years			14–17 years		
Wave 2 (Survey) adjusted^a^	0.005	−0.143 to 0.152	0.949	−0.219	−0.352 to −0.086	0.001
Wave 2 (Survey) adjusted^b^	−0.383	−0.540 to −0.226	<0.001	−0.405	−0.543 to −0.267	<0.001
Female	0.030	−0.043 to 0.103	0.421	−0.025	−0.089 to 0.040	0.450
BMI percentile	0.012	0.011 to 0.013	<0.001	0.010	0.009 to 0.011	<0.001
Height percentile	−0.000	−0.002 to 0.001	0.945	0.001	−0.000 to 0.002	0.126
(Very) good self-rated physical fitness	0.059	−0.024 to 0.141	0.163	0.118	0.041 to 0.195	0.003
RHR (*z*-Score)	0.275	0.239 to 0.312	<0.001	0.271	0.231 to 0.310	<0.001
Birth weight						
Low (<2,5 kg)	0.154	−0.017 to 0.325	0.1355	0.092	−0.049 to 0.232	<0.001
High (≥4,0 kg)	−0.070	−0.187 to 0.048		−0.282	−0.382 to −0.183	
Cuff size						
Medium	0.291	−0.367 to 0.950	<0.001	1.204	0.943 to 1.465	<0.001
Large	−0.176	−0.808 to 0.455		0.861	0.628 to 1.094	
X-Large	−0.386	−1.039 to 0.268		0.704	0.478 to 0.931	

^a^Adjusted only for cuff size.

^b^Adjusted for all covariates.

**Table 3:** Factors associated with mean DBP *z*-score from linear regression models in 3–6-, 7–10-, 11–13-, and in 14–17-year-olds.

Age group	3–6 years			7–10 years		
	Coeff.	95% CI	*p* value	Coeff.	95% CI	*p* value
Wave 2 (Survey) adjusted^a^	0.227	0.096 to 0.358	0.001	−0.051	−0.188 to 0.085	0.459
Wave 2 (Survey) adjusted^b^	0.232	0.112 to 0.353	<0.001	−0.128	−0.267 to 0.010	0.068
Female	−0.019	−0.078 to 0.040	0.533	−0.039	−0.101 to 0.023	0.218
BMI percentile	0.006	0.005 to 0.008	<0.001	0.006	0.005 to 0.007	<0.001
Height percentile	0.010	0.001 to 0.003	0.005	0.001	−0.001 to 0.002	0.307
Sport participation (yes)	−0.092	−0.161 to −0.022	0.010	−0.010	−0.088 to 0.068	0.805
RHR (*z*-Score)	0.289	0.251 to 0.327	<0.001	0.275	0.242 to 0.308	<0.001
Birth weight						
Low (<2,5 kg)	0.120	−0.041 to 0.281	0.161	−0.043	−0.162 to 0.077	0.632
High (≥4,0 kg)	−0.086	−0.214 to 0.042		−0.037	−0.155 to 0.081	
Cuff size						
Medium	−0.535	−0.653 to −0.417	<0.001	−0.907	−1.237 to −0.57	<0.001
Large	−0.729	−0.89 to −0.567		−1.227	−1.569 to −0.885	
X-Large	0.112	−0.246 to 0.470		−1.233	−1.600 to −0.865	

Age group	11–13 years			14–17 years		
Wave 2 (Survey) adjusted^a^	−0.054	−0.206 to 0.098	0.482	−0.166	−0.290 to −0.042	0.009
Wave 2 (Survey) adjusted^b^	−0.330	−0.478 to −0.183	<0.001	−0.290	−0.414 to −0.166	<0.001
Female	0.031	−0.050 to 0.111	0.452	−0.055	−0.119 to 0.009	0.093
BMI percentile	0.006	0.004 to 0.008	<0.001	0.003	0.002 to 0.005	<0.001
Height percentile	0.001	−0.001 to 0.002	0.307	0.001	0.000 to 0.003	0.048
(Very) good self-rated physical fitness	0.028	-0.066 to 0.123	0.556	0.069	−0.004 to 0.142	0.062
RHR (*z*-Score)	0.304	0.266 to 0.342	<0.001	0.315	0.274 to 0.356	<0.001
Birth weight						
Low (<2,5 kg)	−0.016	−0.202 to 0.169	0.366	0.094	−0.048 to 0.236	0.019
High (≥4,0 kg)	−0.103	−0.247 to 0.041		−0.133	−0.244 to 0.021	
Cuff size						
Medium	−0.104	−0.742 to 0.534	<0.001	1.034	0.494 to 1.575	<0.001
Large	−0.490	−1.107 to 0.128		0.669	0.154 to 1.183	
X-Large	−0.682	−1.312 to −0.051		0.644	0.131 to 1.157	

^a^Adjusted only for cuff size.

^b^Adjusted for all covariates.

### RHR changes

Between 2003–06 and 2014–17, the overall mean RHR in children and adolescents (3- to 17-year-olds) has slightly but significantly increased from 82.3 (95% CI 82.0–82.5) to 83.4 bpm (95% CI 82.8–84.0) (*p* < 0.001). Different trends were observed according to the age groups: in 3- to 6-year-olds a slightly not statistically significant decreased (0.6 bpm) was crude observed (Table 1). After adjusting for covariates RHR has statistically significant decreased (Table 4), corresponding to −3.8 bpm over time. In 7- to 10-year-olds no statistically significant changes in the adjusted RHR (in bpm) have been observed; however, in the *z*-score model a small but statistically significant increase has been shown. In 11- to 13-year-olds and in 14- to 17-year-olds a statistically significantly increase by +2.7 and +3.7 bpm, respectively, were observed and this trend was confirmed by adjusting for covariates.

**Table 4:** Factors associated with mean RHR *z*-score from separate linear regression models in 3–6-, 7–10-, 11–17- and 14–17-year-olds.

Age group	3–6 years			7–10 years		
	Coeff.	95% CI	*p* value	Coeff.	95% CI	*p* value
Wave 2 (Survey) crude	−0.061	−0.156 to 0.035	0.211	0.101	0.003 to 0.199	0.043
Wave 2 (Survey) adjusted	−0.122	−0.230 to −0.013	0.028	0.145	0.014 to 0.276	0.030
Female	−0.022	−0.093 to 0.049	0.548	0.021	−0.056 to 0.097	0.595
BMI percentile	−0.004	−0.005 to −0.002	<0.001	−0.003	−0.004 to −0.002	<0.001
Height percentile	−0.004	−0.005 to −0.003	<0.001	−0.002	−0.004 to −0.001	0.004
Sport participation (yes)	−0.097	−0.171 to −0.023	0.011	−0.132	−0.206 to −0.058	0.001
SBP (*z*-Score)	0.268	0.233 to 0.304	<0.001	0.299	0.264 to 0.335	<0.001
Birth weight						
Low (<2,5 kg)	−0.126	−0.279 to 0.028	0.236	0.008	−0.142 to 0.158	0.788
High (≥4,0 kg)	−0.040	−0.159 to 0.080		0.036	−0.067 to 0.139	
Cuff size Medium	0.129	0.020 to 0.237	0.005	0.172	−0.079 to 0.423	0.002
Large	0.179	0.033 to 0.325		0.342	0.086 to 0.598	
X-Large	−0.463	−0.915 to −0.010		0.467	0.171 to 0.763	

Age group	11–13 years			14–17 years		
Wave 2 (Survey) crude	0.204	0.105 to 0.302	<0.001	0.178	0.090 to 0.266	<0.001
Wave 2 (Survey) adjusted	0.284	0.126 to 0.442	0.001	0.351	0.240 to 0.462	<0.001
Female	0.032	−0.056 to 0.119	0.476	−0.028	−0.093 to 0.037	0.395
BMI percentile	−0.003	−0.004 to −0.001	<0.001	−0.004	−0.005 to −0.002	<0.001
Height percentile	−0.001	−0.002 to 0.000	0.204	−0.002	−0.003 to −0.000	0.009
(Very) good self-rated physical fitness	−0.277	−0.365 to −0.189	<0.001	−0.304	−0.371 to −0.237	<0.001
RHR (*z*-Score)	0.296	0.253 to 0.339	<0.001	0.265	0.227 to 0.303	<0.001
Birth weight						
Low (<2,5 kg)	0.042	−0.132 to 0.216	0.877	−0.047	−0.197 to 0.103	0.629
High (≥4,0 kg)	−0.014	−0.143 to 0.116		0.035	−0.071 to 0.141	
Cuff size Medium	0.187	−0.254 to 0.627	0.026	−0.090	−0.716 to 0.536	0.049
Large	0.220	−0.222 to 0.663		−0.080	−0.697 to 0.537	
X-Large	0.372	−0.089 to 0.833		0.061	−0.544 to 0.666	

## Discussion

This large, population-based survey presents changes in mean SBP, DBP, and RHR between 2003–06 and 2014–17 in children and adolescents in Germany. Based on scarce data mean BP in children and adolescents rather seemed to follow decreasing adult BP trends in middle and high-income countries despite the almost ubiquitous pediatric obesity epidemic [4, 12, 26]. Our data confirm the long-term secular trend towards lower mean BP among school-aged children and adolescents. Nevertheless, the increasing BP trend in pre-school children in Germany needs further investigation. Unfortunately, international data on BP trends are from children 8 years and older [12], making it impossible to compare our upward trend in younger children with other publications. Some reports of an inversed BP trend, i.e., of rising BP in children from China, which went along with the rising BP trend in adults in that region, were found. A flattening of the downward SBP trend in children was reported from the US [27, 28] and UK data showed a mean SBP increase by +0.5 mm Hg in 9- to 11-year-old children [16]. Results from a recent meta-analysis showed increasing hypertension prevalence in individuals aged 19 years and younger [29]. We measured BP twice but on a single occasion, which is not comparable with the clinical diagnosis of hypertension. We therefore, do not report the hypertension prevalence in children and adolescents in Germany. Compared to measurements on one occasion the prevalence of hypertension in children and adolescents has been shown to be lower by the factor three to five [30–33].

Many studies have already shown the correlation between overweight/obesity and high BP and that childhood obesity is a strong predictor for future high BP [6, 34]. However, also discordant BP and obesity trends were observed worldwide [12, 26] and BP trends, as in this study, could not be fully explained by BMI trends [9, 35]. Other studies with KiGGS data showed that in this 11 year period overweight and obesity prevalences remained, albeit at a high level, stable across all age groups [36]. Overall, the contribution of BMI and all other high BP risk factors that were available in our study, were small. We could not analyze the full role of changing physical activity patterns since only limited physical activity metrics were comparable for the two time points. In addition, our analyses do not include nutritional factors nor stress. Lifestyle modifications such as increasing physical and a dietary pattern, e.g., according to the DASH-diet (Dietary Approaches to Stop Hypertension), that is high in fruits, vegetables, low-fat milk products, whole grains, fish, and nuts are associated with lower BP [37] could be an underlying reason for improved BP among children and adolescents in Germany, but this could not be investigated.

There are only a few publications from other countries on RHR trends. As far as we know, there was only one population-based study among 9- to 11-year-old-children in the UK (1980–2008), which analyzed changes in RHR and reported an upward trend in this period [3]. The increase in RHR in school children and adolescents in our study could be interpreted as a decreasing physical fitness, and this could be an important parameter for monitoring cardiovascular health in the population. Of note, self-rated physical fitness did not change in our survey over time, but this is a subjective measure prone to a certain degree of reporting bias and not equal to measured fitness. In line with results of the UK study, a recent analysis of KiGGS survey data on physical activity indicate that WHO physical activity recommendation compliance of “at least 60 min of physical activity daily” declined among girls aged 3–10 years in the 5-years period between 2009–12 and 2014–17. Furthermore, the prevalence of “low levels of physical activity” (physical activity of at least 60 min per day on less than 2 days per week) has increased in boys and girls in this period [38]. Nevertheless as in many other studies we could also observe in the regression analysis the strong association between RHR and physical fitness/sport; [39, 40] however, after adjusting for this variable we still observed an increase in RHR over time. Furthermore, a current systematic review on trends in cardio-respiratory fitness among children and adolescents including data from 19 high- and middle-income countries observed a moderate decline in fitness between 1981 and 2014; which is in line with our results [41]. Despite the strong correlation between RHR and SBP [39], BP trends did not parallel RHR trends: in 3- to 6-year-olds SBP and DBP have increased while RHR has decreased; and in the older age-groups BP has decreased while RHR has increased. Currently, we do not have an explanation for this.

Major strengths of our analysis are the large national population database covering a wide age range of children and adolescents (3–17 years) and standardized BP measurements using the same BP device at KiGGS Baseline and at KIGGS Wave 2. A limitation of this study is having only BP measurements on one occasion, which may be a particular limitation for the youngest age group due to inherent difficulties of standardizing measurement aspects such as resting time and sitting position in this age group. Therefore, the observed BP increase and the high proportion of measurements exceeding cutoffs for hypertensive BP await confirmation from other studies. In addition, we have only two time points for interpreting trends and by assessing trends from studies with several points of measure, it is possible to see the fluctuating aspect of trends [4, 27, 35]. Another limitation from our study is the absence of additional covariates (such as food patterns) that could help further explain BP trends or the presence of subjective covariates such as self-rated physical fitness, which does not allow to discard the fact that increasing RHR could be an indicator of decreasing physical fitness. Furthermore, the rule used for cuff selection in both surveys differed; however, we were able to adjust for the cuff size in our analysis. Another challenge that all health examination surveys are facing is the fact that response rates have been declining for decades despite comprehensive methods to encourage participation [18]. The complex schedule of the combined cross-sectional and longitudinal KiGGS Wave 2 survey design further limited scheduling flexibility and may have affected the response. However, weighting factors accounting for age-, sex-, geographic region-, nationality, and education as well as for KiGGS Wave 2 re-participation probability have been applied in order to adjust for differences between respondents and non-respondents.

## Conclusion

BP and RHR trends did not parallel and varied according to the different age groups. Worldwide decreasing mean BP in school children and adolescents, based on far less data than in adults, seemed to resist the obesity epidemic and were reassuring. Increasing BP in younger children, not explained by obesity needs further investigations. In school-aged children and adolescents, the increased RHR trend may indicate decreased physical fitness.

### Summary

#### What is known about this topic

- Blood pressure in children has been decreasing worldwide over decades despite the obesity epidemic but recent data show flattening or reversal of trends.
- The population level of pediatric resting heart rate, a cardiovascular risk, and fitness indicator, is rarely investigated.

#### What this study adds

- Blood pressure and resting heart rate trends in children and adolescents did not parallel and varied according to the different age groups.
- In school-aged children and adolescents, the increased resting heart rate trend may indicate decreased physical fitness.

## Supplementary information

Supplementary Figure 1

Supplementary Figure 2
