# Supplementary figures and images for: Blood pressure and resting heart rate in 3-17-year-olds in Germany in 2003–2006 and 2014–2017
Source: J Hum Hypertens. 2021 Apr 14;36(6):544–53. doi: 10.1038/s41371-021-00535-2 (PMC9225953; doi:10.1038/s41371-021-00535-2)

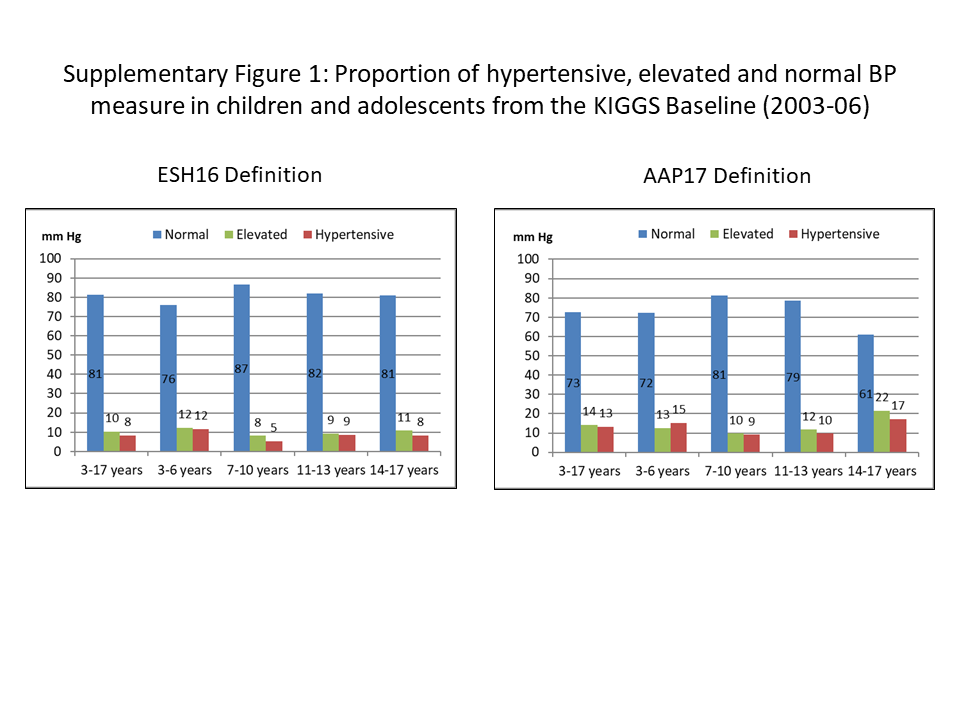

Supplement: Supplementary file 1 — Supplementary Figure 1 [file 41371_2021_535_MOESM1_ESM.tif]

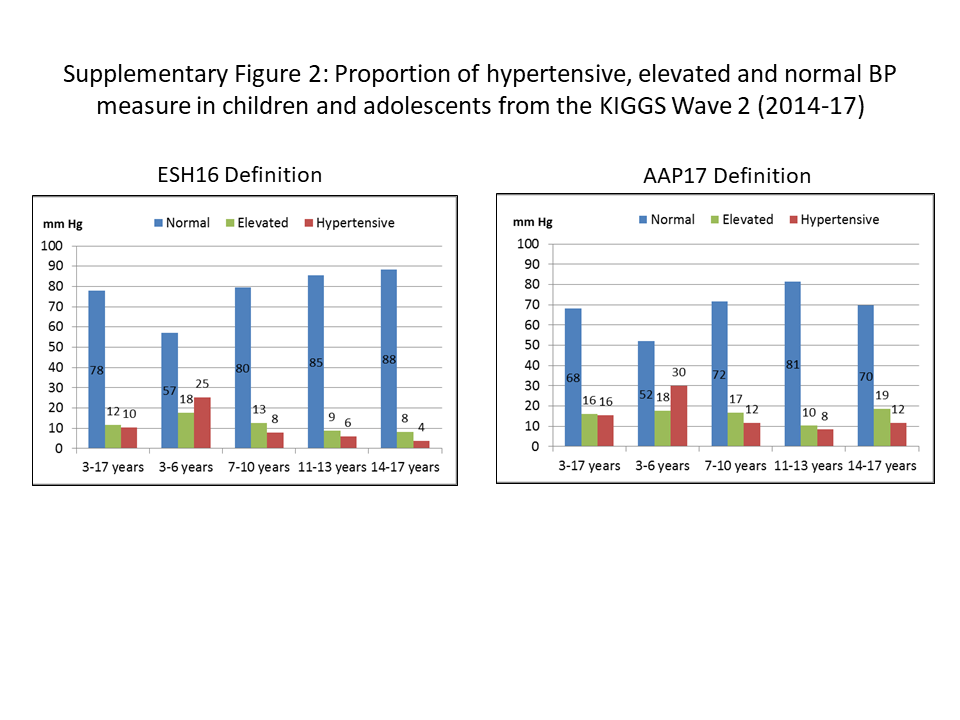

Supplement: Supplementary file 2 — Supplementary Figure 2 [file 41371_2021_535_MOESM2_ESM.tif]
